# Large Area Stress Distribution in Crystalline Materials Calculated from Lattice Deformation Identified by Electron Backscatter Diffraction

**DOI:** 10.1038/srep05934

**Published:** 2014-08-05

**Authors:** Yongliang Shao, Lei Zhang, Xiaopeng Hao, Yongzhong Wu, Yuanbin Dai, Yuan Tian, Qin Huo

**Affiliations:** 1 State Key Lab of Crystal Materials, Shandong University, Jinan250100, P. R. China

## Abstract

We report a method to obtain the stress of crystalline materials directly from lattice deformation by Hooke's law. The lattice deformation was calculated using the crystallographic orientations obtained from electron backscatter diffraction (EBSD) technology. The stress distribution over a large area was obtained efficiently and accurately using this method. Wurtzite structure gallium nitride (GaN) crystal was used as the example of a hexagonal crystal system. With this method, the stress distribution of a GaN crystal was obtained. Raman spectroscopy was used to verify the stress distribution. The cause of the stress distribution found in the GaN crystal was discussed from theoretical analysis and EBSD data. Other properties related to lattice deformation, such as piezoelectricity, can also be analyzed by this novel approach based on EBSD data.

Stress intensity and distribution of crystalline materials seriously affect their properties and the performance of devices based upon them. For example, the bandgap of a compound semiconductor crystal, which determines the emission wavelength of devices, is affected by the stress^1^. Different stress conditions, compressive or tensile, result in different properties. Lattice deformation, represented by changes in the lattice parameters, leads to the introduction of stress^2^,^3^. The stress condition is also important for adjusting crystal growth conditions at different growth stages. So it is important to obtain the stress information of crystalline materials. Various methods have been used to obtain the stress information of crystalline materials. The lattice parameters of crystals can be detected by the X-ray diffraction (XRD) method directly from the angles of diffraction peaks^4^. The stress can be calculated from changes in the lattice parameters between the stressed and stress-free lattice. However, because the radius of the incident X-ray is just several microns, only a small area can be tested by this method. As such, it is difficult to obtain a large area mapping of stress in crystalline materials by XRD. The X-ray microdiffraction method increases the spatial resolution and can be employed to analyze the stress of crystalline materials^5^. High resolution transmission electron microscopy (HR-TEM) can also be employed to analyze lattice parameters directly^6^. However, Just like XRD, it is also difficult to obtain stress distribution information over a large area using HR-TEM. It is also especially challenging to prepare crystalline samples for analysis by HR-TEM. At the same time, several indirect methods have been exploited to analyze the stress condition of crystalline materials. For example, Raman spectroscopy has been used to evaluate the stress of crystalline III-V materials, like gallium nitride (GaN), utilizing the Raman shift wave number distinction of E_2_(high) mode between stress and stress-free GaN crystals^7^. However, the stress value obtained depends on the measurement conditions and the selection of the stress-free GaN crystal E_2_(high) peak position. It is difficult to identify a stress-free crystal accurately. Photoluminescence^8^ and micro-reflectance spectroscopy^9^ have also been employed to analyze the stress of crystalline materials. Stress values obtained by these two methods are generally similar to those obtained from Raman spectroscopy. However, the stress values obtained from these three indirect methods are not acquired from the lattice parameters directly.

The electron backscatter diffraction (EBSD) technique is an effective method to obtain information concerning crystal orientation and for phase identification^10^,^11^. EBSD is also very useful for the microstructural characterization of crystalline materials. Because of the high spatial resolution and good strain sensitivity, EBSD has been used to measure the crystal orientation in compound semiconductor hetero-epitaxial structures^12^,^13^. The distribution of the crystal orientation over a large area can be obtained easily and quickly from EBSD mapping. The orientation data at each point serves as the foundation for analysis of the stress distribution in crystalline materials. With this method, the stress value is obtained directly from the crystal structure because the orientation data given by the geometry of EBSD Kikuchi patterns depends on the crystal structure.

In this article, we report a method for analysis the stress distribution of crystalline materials directly from the lattice deformation derived from the crystallographic orientation identified by the EBSD technique. The method offers a potential to analyze the properties associated with lattice strain. Hexagonal GaN crystal grown by hydride vapor phase epitaxy (HVPE) on a foreign substrate was used as the study sample. The stress distribution of GaN crystal was determined according to the degree of misorientation at different positions of the hetero-epitaxial GaN crystal. The stress conditions obtained by Raman spectroscopy verified the results calculated from the EBSD measurements.

## Experimental

An EBSD system (Oxford Instruments INCA Crystal EBSD system, Nordlys EBSD Detector and HKL CHANNEL5 software) mounted on a field emission scanning electron microscope (FE-SEM, Hitachi S-4800) was used to obtain the strain information of HVPE-grown GaN. The EBSD measurement was operated at 20 kV with a work distance of 20 mm and a sample tilt of 70°.

MOCVD grown GaN on a (0001) sapphire wafer was employed as the substrate for the HVPE process. Patterns on the surface of the MOCVD-GaN were hexagonal mask arrays with diameters of 5.4 μm and a center-to-center distance of 8.1 μm. The patterned substrate was set in a vertical HVPE system with bottom-fed gas after a cleaning process. The thickness of the GaN crystal obtained by HVPE was 430 μm. After a cooling process, the GaN crystal self-separated from the substrate.

Cross-sectional mapping of the free standing HVPE grown in the <0001> direction was conducted by EBSD. The mapping area was 60 μm × 260 μm employing a step length of 1 μm. Z axis mapping by Raman spectroscopy was also employed to identify the stress distribution of the GaN crystal.

## Results

**Figure 1:**
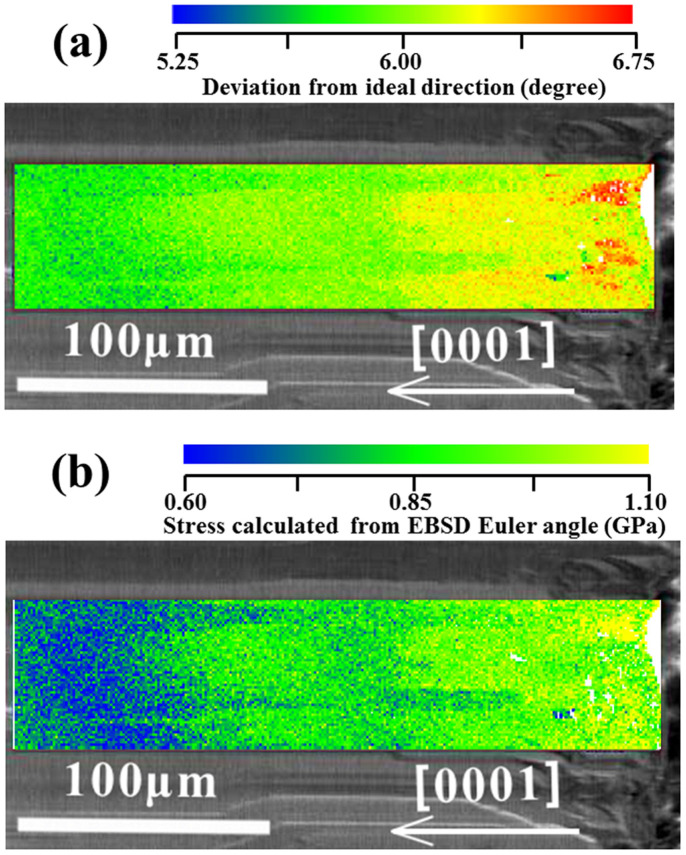
Crystallographic orientations represented as deviations from the <0001> direction obtained from EBSD mapping data (a), and the calculated stress values based upon the EBSD crystallographic orientation mapping results (b).

EBSD mapping of a cross-section of GaN crystal was obtained, as shown in Fig. 1 (a). The <0001> direction was set as the reference direction. Deviation from the <0001> direction was obtained from the spatial orientation information collected from backscattered Kikuchi diffraction patterns (three Euler angles) at each EBSD mapping point. The EBSD results show that the deviation from the <0001> direction near the interface is larger than that far from it. The mapping color near the interface is yellow and red, which indicates a deviated from the <0001> direction of 6.2°. As the distance from the interface increases, the mapping color changes from yellow to green and the deviation from the <0001> direction reduces to 5.8°. This result demonstrates that the extent of misorientation near the interface is greater than that far from the interface and deviation from the <0001> direction decreases with increasing distance from the interface.

## Discussion

In crystalline materials, mechanical stress and deformation are, within the limit of Hooke's law, proportional to one another. For sufficiently small stress and deformation, the relationship of these quantities can be described, when infinitesimal torques or rotations are neglected^14^, by 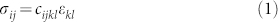
In equation (1), *c_ijkl_* is the elasticity tensor whereby the stress tensor (*σ_ij_*) can be calculated from the deformation tensor (*ε_kl_*). The deformation is described by changes in the lattice parameters between the stressed and stress-free lattice. The elasticity tensor is a fourth-rank tensor with 3^4^ = 81 components, although symmetry reduction decreases the number of independent components.The number of independent components and, hence, the expression of *c_ijkl_* vary according to different crystallographic symmetry conditions. The stress can be calculated by this relationship using a material-specific elasticity tensor.

Single crystal GaN, which is a wurtzite structure crystal, possesses C_6v_^4^ space-group symmetry, and is an example of a hexagonal crystal system. The stress distribution of GaN crystal on a foreign substrate is apparent. The GaN crystal was grown on a sapphire substrate along the <0001> direction. However, the actual growth direction deviated slightly from the <0001> direction due to the mismatch of the lattices and thermal expansion coefficients between GaN and the sapphire substrate. Stress was introduced because of the resulting misorientation^15^,^16^. Dislocations and cracks released some of the stress local to the defect, but residual stress remained in the GaN crystal. The distribution of the stress along the <0001> direction indicate that the stress decreased with increasing distance from the original interface between the GaN crystal and the substrate (N polar face), and the smallest value of stress was observed at the top free surface of the GaN (Ga polar face)^7^. The stress distribution is distinct in this kind of hetero-epitaxial crystal. In this case, EBSD mapping is appropriate for stress calculation of the GaN crystal grown on a foreign substrate.

**Figure 2:**
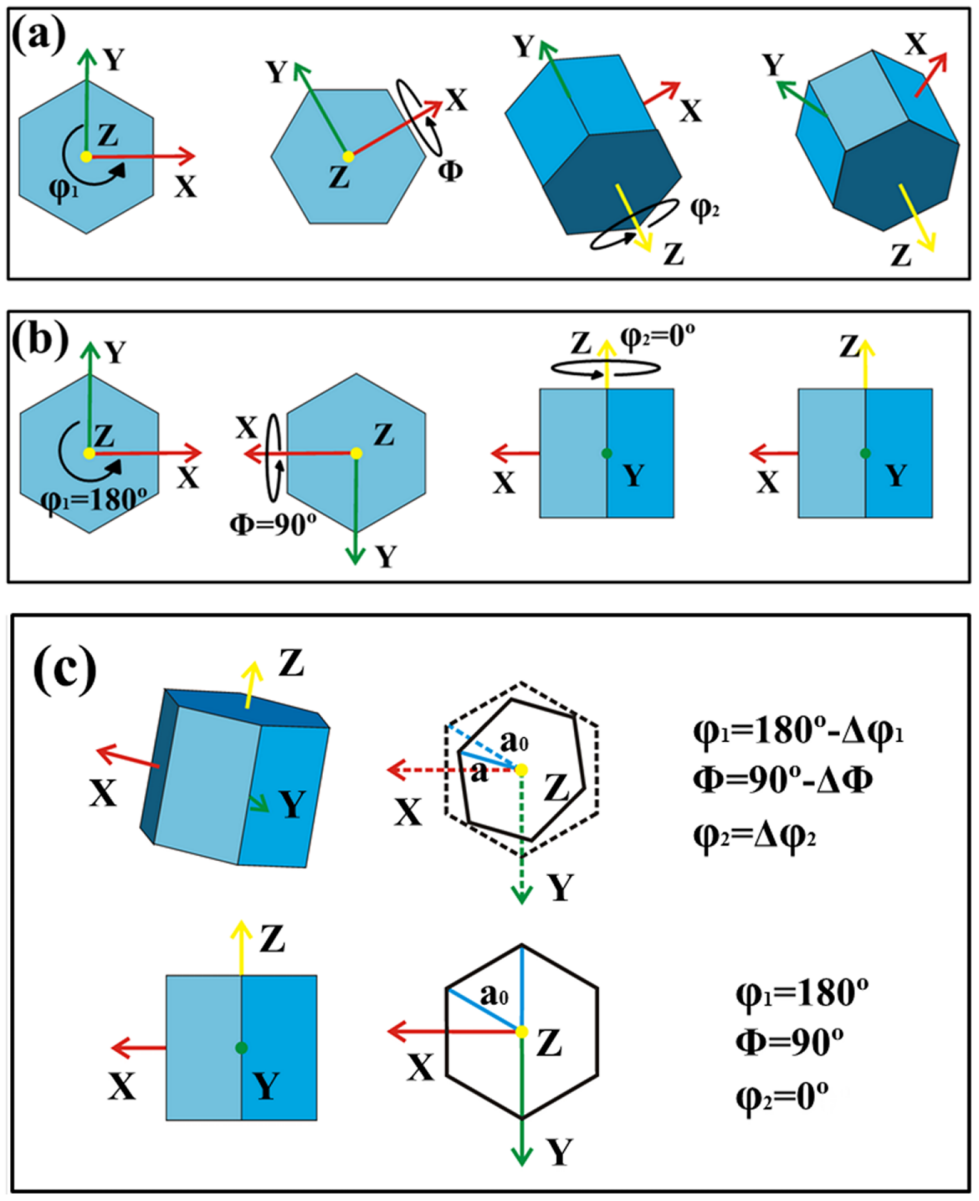
Euler angle rotations according to Bunge's convention (a), and ideal Euler angle values of a cross-section of free-standing GaN crystal self-separated from the sapphire substrate along the <0001> direction (b). Comparison of the lattice projections on the (0001) plane for the ideal and actual crystallographic orientations (c).

EBSD mapping was used to determine the crystallographic orientation at regular points on the sample for calculation of the stress. In EBSD mapping, the crystallographic orientation is recorded using three Euler angles that describe a minimum set of rotations that can bring one orientation into coincidence with another. During a measurement, this is the relationship between the EBSD detector and the particular point on the sample being measured^17^. Bunge convention is most commonly used to describe the Euler angle rotations. The three Euler angles (φ_1_, Φ and φ_2_) represent the following rotations, which are shown schematically in Fig. 2(a): first, a rotation of φ_1_ around the Z-axis; second, a rotation of Φ around the rotated X-axis; and third, a rotation of φ_2_ around the rotated Z-axis. The ideal Euler angle values for a cross-section of a GaN crystal grown on a sapphire substrate along the <0001> direction are φ_1_ = 180°, Φ = 90° and φ_2_ = 0° as shown in Fig. 2(b). In this case the cross-section surface of the GaN crystal is identified as (11–20) in the hexagonal crystal system. However, the actual crystallographic orientation of GaN deviates from the ideal value, as shown in Fig. 2(c).

Misorientation of the GaN crystal was determined by the differences between the ideal Euler angles and the actual values. The EBSD mapping data show that the three Euler angles of GaN deviated from the ideal values. These deviations are given as: 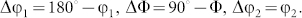


The angle deviations indicate the degree of misorientation, and they affected the lattice parameter at the projections on the (0001) plane (Fig. 2 (c)). The lattice parameters of the projection can be calculated from the rotation matrix. The relationship between the Bunge-Euler angles and the rotation matrix^18^ is 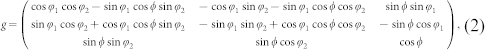
which can be described in terms of the misorientation angles as: 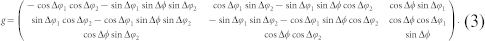
The lattice parameter is determined as the distance from the origin point to point 
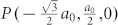
 in the initial coordinates. Based upon the ideal condition (φ_1_ = 180°, Φ = 90° and φ_2_ = 0°), the *P* rotates to point 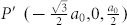, indicating that the c-plane moves to the XOZ plane in the initial coordinates. Taking the misorientation into account (using equation (3)) point P rotates to point *P″* according to the following: 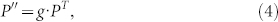
where *T* represents the transpose, resulting in 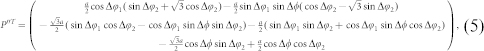
The XOZ plane is set as the basis plane, and in this case, using the projection of *P″* on the XOZ plane, the lattice parameter is 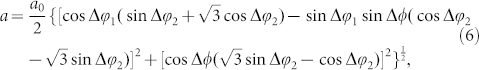
where *a* is the actual lattice parameter and *a_0_* is ideal value.

To the space-group symmetry of single crystal GaN, the strain tensor *ε* is diagonal and possesses the components: 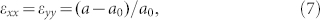

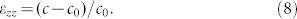


In these equations, *a* and *c* are the lattice parameters of single crystal GaN, where *a_0_* and *c_0_* are the lattice parameters in a condition of equilibrium. The corresponding diagonal stress tensor components are given by Hooke's law in the limit of small deviations from equilibrium as 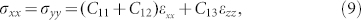

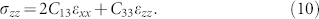


Four of the five independent stiffness constants *C_ij_* of the considered wurtzite crystal using in equation (9) and equation (10) are shown in Table 1^19^,^20^. The biaxial stress in the plane perpendicular to the c axis of the lattice is described by constant forces in this plane such as 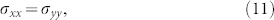

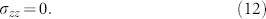


Then, Hooke's law (equation (9) and equation (10)) provides a relationship between the strain components is 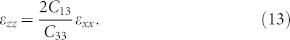
The biaxial modulus is given in terms of the elastic stiffness constants as 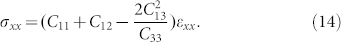
The relationship between the stress and strain is obtained from equation (14).

The stress obtained via equation (7) by EBSD (from equation (6)) is 
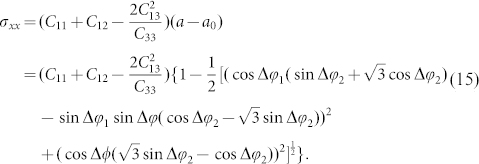
The stress obtained from equation (15) is shown in Fig. 1 (b), the stress value is large near the interface, decreases towards the middle region, and, approaching the Ga-polar face, the stress becomes small and constant.

**Figure 3:**
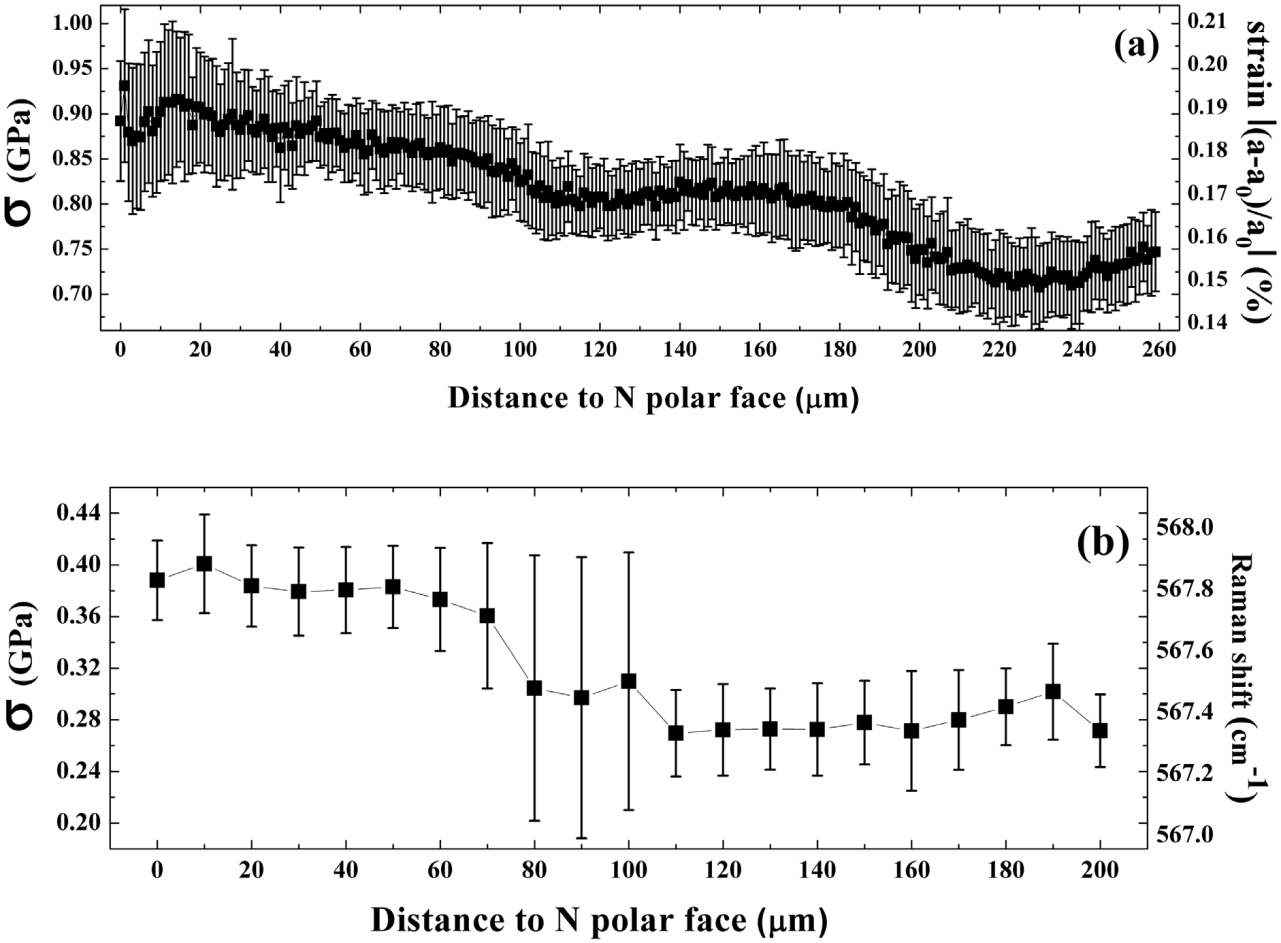
The stress values calculated the crystallographic orientations at different positions in the cross-section of the free-standing GaN crystal with respect to the distance from the N-polar face (a), and the E_2_(high) Raman mode peak position at different distances from N polar face and the corresponding stress (b).

Raman spectroscopy was also carried out to evaluate the stress distribution in the hetero-epitaxial GaN crystal. For wurtzite structure GaN, E_2_(high) mode of Raman spectroscopy is considered to be affected only by stress. A biaxial stress of one GPa shifts the E_2_(high) Raman mode by 4.2 cm^−1^, and the wave number of the stress-free GaN E_2_(high) mode^21^ was taken as 566.2 cm^−1^. The free-standing GaN crystal self-separated from the substrate was measured by Raman spectroscopy at different depths from the N-polar face towards the Ga-polar face along the <0001> direction. The peak position of E_2_(high) Raman mode at different depths and the corresponding stress are shown in Fig. 3 (b), where a depth of 0 μm represents the surface of the N-polar face, and increasing distance indicates motion towards the Ga-polar face. The peak position of E_2_(high) Raman mode near the interface (0–70 μm) exhibits a higher wave number than it far from the interface (110–200 μm). These results demonstrate that the stress was larger near the interface than near the free surface, and a middle transition region (70–110 μm) is observed.

The stress distribution of the GaN crystal obtained from Raman spectroscopy is consistent with the results calculated from EBSD data. Both the results obtained from these two methods demonstrate that the stress near interface is large, obviously decreases over a middle range, and maintains a small value over some distance from the Ga-polar face. Clearly, the impact of the sapphire substrate on the stress of the GaN crystal occurs over a limited range of distance. As such, the impact becomes a small and constant for a sufficiently thick GaN crystal because the lattice parameter approaches a value close to that of the stress-free condition sufficiently far from the interface. Therefore, the lattice mismatch described by Equation (6) also becomes small. It should be noted that the stress value calculated from EBSD is larger than that obtained from Rama. This is because the stress value obtained from Raman is a relative value that depends on the selection of the E_2_(high) mode wave number associated with a stress free crystal. However, it is difficult to identify a point on the crystal surface that is completely stress-free. Therefore, the stress determined by empirically selecting the E_2_(high) mode wave number of a stress-free crystal is not absolutely accurate. However, the influence of different kinds of stress on the EBSD data is not the same. The calculated stress will be underestimated when based on misorientation in some condition. Meanwhile, other properties related to lattice deformation, such as piezoelectricity, can also be analyzed by this approach based on EBSD data. The only difference is that the elasticity tensor will be replaced by some other tensor. The good sensitivity makes analysis on a micron scale convenient, at the same time; properties distributed over a large area can also be obtained by this method.

## Conclusions

A method for calculating stress in crystalline materials directly using the lattice deformation identified by EBSD was provided. The stress of crystalline materials at each mapping point was obtained from EBSD by this method. The stress distribution over a large area was obtained efficiently and accurately. Crystalline GaN of a wurtzite structure grown by HVPE on a foreign substrate was used as an example of a hexagonal crystal system. The stress distribution of the GaN crystal obtained from Raman spectroscopy confirmed the results provided by EBSD. We believe that the stress distributions of other crystalline materials can also be calculated using this method simply by changing the form of the elasticity tensor.

## Author Contributions

X.H. and Y.W. designed experiment. Y.S. wrote the main manuscript text and carried out EBSD measurements. L.Z., Y.D., Y.T. and Q.H. grew the sample. All authors reviewed the manuscript.

## Figures and Tables

**Table 1:** Elastic constants (GPa) of wurtzite GaN crystal

	GaN(Wz)
C_11_	390^19^
C_12_	145^19^
C_13_	106^20^
C_33_	398^19^
